# Fine mapping and functional study of the systemic lupus erythematosus-associated *NMNAT2/SMG7 *locus

**DOI:** 10.1186/ar4627

**Published:** 2014-09-18

**Authors:** Jian Zhao, Yun Deng, Jennifer M Grossman, Betty P Tsao

**Affiliations:** 1 David Geffen School of Medicine University of California, Los Angeles, CA, USA

## Background

*NMNAT2 *(rs2022013 located at intron 1) was identified as a SLE risk locus in a European-derived population in a genome-wide association study (GWAS). *NMNAT2 *(nicotinamide mononucleotide adenylyltransferase 2) is expressed mainly in the brain, regulating energy metabolism. Proximal to the SLE-associated *NMNAT2 *variant is *SMG7*, encoding a component of the mRNA quality control pathway that regulates spliceosomal machinery such as Sm and snRNP via alternative splicing. We fine mapped the *NMNAT2/SMG7 *region in multiple ancestries and explored functional consequences of the identified variants.

## Materials and Methods

We genotyped/imputed 313 SNPs covering an ~550 kb *NMNAT2/SMG7 *region in 15,424 case-control subjects from European-Americans (EA), African Americans, Asians and Amerindian/Hispanics, assessed SNPs for association with SLE using a logistic regression model adjusted for sex and ancestry, and used haplotype-based conditional testing to distinguish independent associations. Quantitative real-time PCR and luciferase reporter assays were used to examine allelic differences in *SMG7 *expression and transcription activity. PBMCs from SLE patients (*n *= 13) were cultured with or without siRNA targeting *SMG7, GAPDH *(positive control) or siRNA with a nontargeting sequence (NC, negative control), and culture supernatants were measured by ELISA for levels of antinuclear antibody (ANA) and cytokines/chemokines.

## Results

We confirmed association at rs2022013 and identified two independent signals in EA only: intron 1 of *NMNAT2 *tagged by rs12146097 (*P *= 1.5 × 10^-10^, OR = 1.38); and multiple *SMG7 *SNPs tagged by rs2275675 (*P *= 5.7 × 10^-8^, OR = 1.22). Expression quantitative trait locus data showed SLE-risk alleles of *NMNAT2/SMG7 *variants consistently associated with decreased mRNAs of *SMG7*, but not *NMNAT2*, in cell lines, suggesting *SMG7 *is a more likely risk gene for SLE. The rs2275675 risk allele was associated with decreased *SMG7 *mRNAs dose dependently in PBMCs of 86 SLE patients and 119 controls (*P *= 0.001 and 6.84 × 10^-8^, respectively), and reduced transcription activity in two transfected cell lines (*P *≤ 0.004). *SMG7 *mRNA levels in PBMCs correlated inversely with ANA titers in 68 SLE patients (*P *= 0.0089, *r *= -0.31). Compared with culture supernatants of SLE PBMCs treated with NC-siRNA, those treated with *SMG7-*siRNA showed increased ANA (*P *< 0.0001) and CCL19 (*P *= 0.0002; a ligand for CCR7 promoting movement/ interaction of B-Th cells and antibody production).

## Conclusions

We confirmed the previous GWAS *NMNAT2 *association and identified independent *SMG7 *association with SLE in an EA population. The SLE-risk alleles are dose-dependently associated with decreased *SMG7 *mRNAs, and *SMG7 *reduction increases ANA and CCL19 production in PBMC cultures of SLE patients, suggesting that dysfunction in mRNA surveillance conferred by SLE-associated *SMG7 *variants contributes to SLE manifestations.

